# Consensus on the Development of Vaccines against Naturally Acquired Melioidosis

**DOI:** 10.3201/eid2106.141480

**Published:** 2015-06

**Authors:** Direk Limmathurotsakul, Simon G.P. Funnell, Alfredo G. Torres, Lisa A. Morici, Paul J. Brett, Susanna Dunachie, Timothy Atkins, Daniel M. Altmann, Gregory Bancroft, Sharon J. Peacock, for The Steering Group on Melioidosis Vaccine Development

**Affiliations:** Faculty of Tropical Medicine, Mahidol University, Thailand (D. Limmathurotsakul);; Public Health England, London, United Kingdom (S.G.P. Funnell);; University of Texas Medical Branch, Galveston, Texas, USA (A.G. Torres);; Tulane University School of Medicine, New Orleans, Louisiana, USA (L.A. Morici);; University of South Alabama, Mobile, Alabama, USA (P.J. Brett);; University of Oxford, Oxford, United Kingdom (S. Dunnachie);; Defence Science and Technology Laboratory, Porton Down, United Kingdom (T. Atkins);; Imperial College, London (D. M. Altmann);; London School of Hygiene and Tropical Medicine, London (G. Bancroft);; University of Cambridge, Cambridge, United Kingdom (S.J. Peacock)

**Keywords:** *Burkholderia pseudomallei*, melioidosis, Whitmore’s disease, bacteria, vaccine, antimicrobial drugs, prevention, Steering Group on Melioidosis Vaccine Development, intraperitoneal, intravenous, aerosol inhalation, intranasal, challenge dose

## Abstract

Several candidates for a vaccine against *Burkholderia pseudomallei*, the causal bacterium of melioidosis, have been developed, and a rational approach is now needed to select and advance candidates for testing in relevant nonhuman primate models and in human clinical trials. Development of such a vaccine was the topic of a meeting in the United Kingdom in March 2014 attended by international candidate vaccine developers, researchers, and government health officials. The focus of the meeting was advancement of vaccines for prevention of natural infection, rather than for protection from the organism’s known potential for use as a biological weapon. A direct comparison of candidate vaccines in well-characterized mouse models was proposed. Knowledge gaps requiring further research were identified. Recommendations were made to accelerate the development of an effective vaccine against melioidosis.

Melioidosis vaccines are urgently needed for both public health and biodefense purposes. The bacterium *Burkholderia pseudomallei*, the causal organism of melioidosis, was designated as a category B select agent by the US Centers for Disease Control and Prevention (CDC) and as a Tier 1 select agent by National Select Agent Registry (NASR), a joint program of the CDC and the Animal and Plant Health Inspection Service of the US Department of Agriculture. These designations have led to considerable research funding to develop a melioidosis vaccine (*1*,*2*). The potential for humans to be infected with *B. pseudomallei* by inhalation, the low infective dose by this route, and difficulties associated with diagnosis and treatment have led to this organism being considered to be at high risk for deliberate misuse as a weapon (*3**,**4**,**5*). 

*B. pseudomallei* is readily isolated from soil and water in many tropical areas across Southeast Asia, the Indian subcontinent, northern Australia, and parts of South America and the Caribbean (*6*). In these areas, melioidosis can be naturally acquired by skin inoculation, ingestion, and inhalation of environmental *B. pseudomallei*. In northeast Thailand, melioidosis kills an estimated >1,000 persons per year (*7*). A vaccine could be cost-effective for the prevention of melioidosis among populations at high risk for infection by *B. pseudomallei*, such as persons who have diabetes (*8*).

Establishing a process for selecting, assessing, and advancing potential vaccine candidates is a critical issue for the melioidosis research community. Several vaccine candidates have been shown to provide partial protection in murine models of infection (*8*–*10*), but none have been tested to date in nonhuman primates (NHP) or humans. Possible candidates include live attenuated, whole-cell killed, subunit, glycoconjugate, outer membrane vesicle, plasmid DNA, and dendritic cell vaccines (*8*); possible candidates and candidate developments have been systematically reviewed and reported (*8*–*10*). A notable lack of standardization of protocols has resulted in variability in the animal models used (mice [BALB/c, C57BL/6, and Porton] and Syrian hamster); route of inoculation (intraperitoneal, intravenous, intranasal, intradermal, and subcutaneous); challenge strains (K96243, 1026b, 576, NCTC13392, NCTC4845, and NCTC13179); route of *B. pseudomallei* challenge (intraperitoneal, intravenous, aerosol inhalation, and intranasal); challenge dose; and duration of the follow-up period for assessing mortality rates among acute and chronic challenge models (5 days to 5 months) (*8*–*10*). As a result of the lack of consistency in use of specific methods on specific models, it is not possible to determine the comparative efficacy of different vaccine candidates. This inconsistency prevents researchers and officials in sponsoring and funding agencies from deciding which candidates should be advanced to NHP models. Recognizing these problems, our objectives were to assemble an expert group and arrive at consensus opinions and recommendations on candidate vaccines and animal models based on currently available evidence (*8*–*10*).

## The Working Group

The Steering Group on Melioidosis Vaccine Development (SGMVD) was conceptualized during attendance of the VIIth World Melioidosis Congress (WMC), held in Bangkok, Thailand in September 2013. A questionnaire was initially composed by D.L. and S.J.P. based on perceived uncertainties and was intended to generate discussions regarding these barriers to progress (online Technical Appendix, http://wwwnc.cdc.gov/EID/article/21/6/14-1480-Techapp1.pdf). The questionnaire was circulated to all members of the steering group in October 2013. Responses were collated and recirculated to all members during January 2014. All members of SGMVD returned the questionnaire. Then, additional experts from the United Kingdom and United States were invited to a face-to-face meeting of the steering group, which was held in March 2014 at Public Health England (PHE), Porton Down, United Kingdom. All members except Bart Currie attended the face-to-face meeting. Recommendations from the SGMVD were circulated for approval. All members agreed with the consensus opinions of the meeting attendees.

## Development of Melioidosis Vaccines to Prevent Natural Infection Versus Biodefense Use

The development process of a melioidosis vaccine that prevents naturally acquired infection is likely to be different than that for biodefense purposes (Table). This difference is because a biodefense vaccine is intended to protect healthy persons from inhalational inoculation, whereas a vaccine against natural infection will be required to protect immune-compromised hosts, such as persons with diabetes, who comprise the highest risk group and are most often infected by skin inoculation (*8*). Therefore, the route of challenge in animal models should differ: inhalation should be the focus for biodefense vaccine development, and subcutaneous inoculation for natural infection vaccine development. It is also important to consider whether diabetic mouse models should be included for the evaluation of public health vaccines, and if so, which of the available diabetic mouse models best reflects the increased susceptibility of the at-risk human population. An additional consideration is whether postexposure prophylaxis with antimicrobial drugs should be included in models for biodefense vaccines. Sharing of information between different groups would enhance progress. Because the development of biodefense vaccines and therapeutics is already supported by other groups (e.g., Defense Threat Reduction Agency, National Institute of Allergy and Infectious Disease, and Biomedical Advanced Research and Development Authority [BARDA]), this process was not discussed further by the group (*8*–*11*). The SGMVD agreed to focus further discussions on melioidosis vaccines that prevent naturally acquired infection.

**Table:** Comparison of potential usage and animal models required for development of vaccines against naturally acquired melioidosis versus melioidosis vaccines for biodefense purposes*

Vaccine characteristics	Vaccines against naturally acquired melioidosis	Melioidosis vaccines for biodefense purposes
Target population		
Character	Persons with diabetes mellitus	Healthy persons
Route of exposure	Skin inoculation	Inhalation
Potential use of vaccine		
Prophylaxis	Yes	Yes
Prophylaxis plus postexposure antimicrobial drug administration	Yes*	Yes†
Animal models		
Addition of diabetic mouse model	Required	Not required
Route of *Burkholderia pseudomallei* challenge	Subcutaneous	Inhalation
Nonhuman primate model	Required	Required

*Administration of antimicrobial drugs after symptoms occur. †Administration of antimicrobial drugs after release.

## Potential Use of Melioidosis Vaccines to Prevent Natural Infection in Melioidosis-Endemic Areas

During a discussion of needs for vaccines against naturally acquired melioidosis, steering group member Charung Muangchana, director of National Vaccine Institute (NVI), Ministry of Public Health, Thailand, supported the public health need for a melioidosis vaccine in Thailand. The point was made that no specific exclusion criteria would be proposed at the outset by NVI; for example, killed whole-cell vaccines should not be excluded as a potential melioidosis vaccine to be used for public health purposes in Thailand. A killed whole-cell vaccine could be considered if it passed phases I, II, and III clinical studies; had minimal side effects; and proved to be a cost-effective intervention in Thailand.

None of the candidate vaccines tested to date have provided sterilizing immunity, but it was proposed during discussion that this should not be a barrier to using the best candidates in trials using NHP models. A melioidosis vaccine could be a cost-effective intervention for public health in areas to which melioidosis is endemic even if it only provided partial protection, resulting in reduction in disease severity and death rates among infected persons (*8*). For example, a melioidosis vaccine that reduced the incidence of melioidosis by 25%, reduced the case-fatality rate by 25%, provided protection for 3 years, and cost <100 Thai Baht (≈$3 US) per course would be cost effective for a high-risk population in Thailand (estimated for population of persons who have diabetes, at a baseline annual incidence rate of 150 cases per 100,000 persons per year, and a fatality rate of 40%) (*8*).

## Process of Candidate Selection

The SGMVD recommended that the approach used to select, develop, and clinically evaluate tuberculosis (TB) vaccine candidates (*12*) be used as an example for a gating process for melioidosis candidate selection, because the challenges of vaccine development for TB and melioidosis are similar. No melioidosis vaccine has been tested in humans, and much could be learned from the TB vaccine testing process. The SGMVD agreed that a head-to-head comparison of candidate vaccines in standardized mouse models would support decision making by developers, sponsors, funders, and policy makers concerning the choice of candidate vaccine(s) advanced for testing in NHP models. To reduce any potential bias in candidate selection and conflicts of interest, SGMVD agreed that the head-to-head comparison should be performed in an institution that is independent of the vaccine developer. The gateway system and background information requested is similar to those features in the process of candidate selection of other vaccines (*12*). The candidate vaccine developer will also be responsible for the preparation of a trial lot to be tested at an independent institute (*12*). These processes are similar to that being conducted for TB vaccine candidate evaluation (*12*).

The SGMVD noted that the information obtained from the head-to-head mouse model may not necessarily reflect the outcome that will be seen in a NHP model and eventually in human clinical trials. The purpose of using a combination of mouse and NHP models is to carefully select the optimal candidate vaccines for future clinical studies.

## Standardized Mouse Models for Head-to-Head Vaccine Candidate Comparison

### Mouse Models

The SGMVD recommended that 2 mouse models (BALB/c and C57BL/6) be used for the head-to-head candidate vaccine assessment. These models were selected because they exhibit differential susceptibilities to *B. pseudomallei* infection: C57BL/6 mice are more resistant (*13*,*14*). The evaluation should focus on protection from the acute stage of infection in both mouse models, reflecting the need to protect humans from rapidly fatal disease.

The SGMVD recommended that a diabetic mouse model should also be considered for use during the head-to-head vaccine assessment. However, inherent problems were recognized, and a specific diabetic mouse model was not selected because of the current paucity of information on melioidosis in diabetic mouse models.

### Formulas, Dosages, and Routes of Immunization

The SGMVD agreed that the production of the candidate vaccine, formulation, adjuvants used, toxicity, immunogenicity, recommendations for dosage, and routes of immunization are the responsibility of the developers. During the head-to-head comparison, the study institute should administer the candidate vaccine according to the guidelines provided by the developers.

### Control Groups

The SGMVD recommended the use of 2 control groups in each head-to-head comparison: a negative-control nonvaccinated group and a positive-control immunized group. The nonvaccinated group acts as a control for mortality outcome. The positive-control immunized group provides a benchmark for partial protection of a known vaccine candidate. The live attenuated mutant *B. pseudomallei ilvI* (commonly referred to as 2D2) is proposed as a positive control for the positive-control immunized group on the basis of high efficacy of 2D2 (*15*,*16*). This method has been tested in 2 independent laboratories in the United Kingdom (*15*,*16*), is effective in both genetically susceptible (BALB/c) and resistant (C57BL/6) mouse strains, and is one of the most protective vaccines to be tested in the *Burkholderia* research field. It is easy to prepare as a standardized batch reagent (albeit under biosafety level 3 conditions) for use as a reference for the effectiveness of other vaccine candidates. Although this method has not been tested for efficacy against a wide variety of *B. pseudomallei* strains, it would be predicted to provide cross-protection on the basis that it is a live attenuated vaccine and will be expressing a range of antigens in the host. Confirming this efficacy against a chosen panel of approved *B. pseudomallei* challenge strains represents a crucial and feasible first step for its inclusion as a reference standard in future vaccine development programs.

### Challenge Strains of *B. pseudomallei*

The SGMVD proposed the use of 2 or 3 bacterial strains selected from a standard set of *B. pseudomallei* isolates characterized and recommended by BARDA for head-to-head vaccine candidate comparisons, rather than a single randomly selected strain (*11*). This choice was made because there is clear evidence for strain-to-strain variation in virulence of *B. pseudomallei*, which can be further confounded by the number of strain passages and quality of bacterial storage. Six strains are recommended for initial consideration: MSHR668, MSHR305, 1026b, 1106a, K96243, and 406a. Each of the 6 strains prepared by BARDA has extensive and complete information, including source, passage history, virulence, genotype, and phenotype, and all are available from BARDA (*11*). Refining the recommendation for a subset of these 6 strains could be made after more data from mouse models are available.

### Challenge Route of *B. pseudomallei*

To replicate the most common route of natural melioidosis infection in humans, the SGMVD suggested that the subcutaneous route be used for the challenge dose of *B. pseudomallei*. Current knowledge on mouse models challenged via the subcutaneous route of *B. pseudomallei* is limited (*17*), and further studies are required to provide baseline data. No vaccine candidate to date has been evaluated for efficacy against subcutaneous inoculation with *B. pseudomallei* (*8*–*10*). The SGMVD proposed that baseline information should be generated for each of the 6 strains recommended by BARDA in BALB/c and C57BL/6 mice by using the subcutaneous route.

### Challenge Dose of *B. pseudomallei*

Because the aim of the head-to-head comparison is to compare the efficacy between >2 vaccine candidates, the SGMVD proposed that the challenge dose of *B. pseudomallei* be adjusted so that all animals in the negative-control nonvaccinated group reach humane euthanasia endpoints at 7–14 days postchallenge. If the challenge dose of *B. pseudomallei* is too high, both groups could reach euthanasia endpoints too quickly, and the protective efficacy in vaccinated groups may not be evident. This practice was observed in a study evaluating vaccine candidates in protection against airborne challenge, in which all animals in the negative-control (nonvaccinated) group died by day 4 and all animals in the vaccinated groups died by day 5 (*18*). Alternatively, if the challenge dose of *B. pseudomallei* is too low, only a fraction of the nonvaccinated control groups may reach euthanasia endpoints, and the power to detect protective efficacy in vaccinated groups will be very low in a survival analysis. This result has been commonly observed (*8*–*10*) when bacterial burden of major organs has been used as a secondary endpoint to evaluate protection from subclinical infection.

### Timing of Challenge

It is essential that the *B. pseudomallei* challenge be done no sooner than 4 weeks after the last dose of vaccine to enable residual innate immune responses to quiesce. Otherwise, the survival benefit observed in mouse models could be overestimated because of the residual short-term innate immune response rather than the adaptive immune response induced by the vaccine.

### Duration of Follow-up for Mortality Rate Outcome

The SGMVD proposed that the duration of follow-up efforts should be sufficient to compare the efficacy between the candidates. The steering group suggested a follow-up period of at least 28 days after challenge.

## Areas Requiring Further Advancement

### Animal Melioidosis Models Using Subcutaneous Route

Much remains to be learned regarding use of subcutaneous *B. pseudomallei* inoculation in animal models. Most of the animal models developed in the past used intraperitoneal, intravenous, or inhalational *B. pseudomallei* inoculation; only a small number used a subcutaneous route (*17*,*19*–*21*). A study using goats to compare subcutaneous versus intraperitoneal inoculation showed that, in animals inoculated intraperitoneally, septicemia with multiple microabscesses developed throughout the body, whereas in animals inoculated subcutaneously, localized abscesses developed in the lungs and spleen (*22*). It has been shown that the dose of *B. pseudomallei* required to result in a rapidly fatal infection administered by the subcutaneous route in mice is considerably higher than that required when using the inhalational route (*17*). The Defence Science and Technology Laboratory (Dstl), United Kingdom, has conducted subcutaneous *B. pseudomallei* challenge in marmosets (*Callithrix jacchus*) (*23*), but a 50% lethal dose could not be estimated because all 14 marmosets died within 4 days of infection when the lowest dose of 26 CFU was used (*23*). The study shows that most marmosets challenged with subcutaneous inoculation have a systemic disease with occasional lung involvement (*23*).

### Diabetic Murine Model

A small number of diabetic mouse models have been evaluated with *B. pseudomallei* infection, but none of the candidate vaccines have been evaluated in diabetic mice. Although streptozotocin-induced diabetic mice have been used to study a range of diseases, in 1996, Brett and Woods reported that adult streptozotocin-induced diabetic Sprague-Dawley rats were not susceptible to intraperitoneal challenge with *B. pseudomallei* (*24*). In 2011, Hodgson et al. reported the use of 8-week-old BKS.Cg-*Dock7^m^* +/+ *Lepr^db^*/J mice that carried a genetic mutation in the leptin receptor (*db/db*) and become insulin resistant, diabetic, and hyperglycemic (*24*). The study showed that diabetic mice died more rapidly than nondiabetic mice after subcutaneous challenge with *B. pseudomallei* and that macrophages from diabetic mice were unable to contain and kill *B. pseudomallei* (*20*). In 2013, Hodson et al. compared C57BL/6J-*Dock7^m^ Lepr^db^*/++ mice to diet-induced C57BL/6 diabetic mice and found that the metabolic profiles of the 2 models were not different. The study also showed that diet-induced diabetic mice died more rapidly than did nondiabetic mice after subcutaneous challenge with *B. pseudomallei* and that cytokine responses were impaired in the diabetic mice (*21*). It has been suggested that diet-induced C57BL/6 diabetic mice might be better models than diabetic homozygotes because *db/db* mice exhibit additional immune dysfunctions when compared to type 2 diabetic mice induced by a high-fat diet (*21*). Nonetheless, several other current and upcoming mouse models use more sophisticated strategies for gene inactivation that more closely replicate insulin resistance in type 2 diabetes (*25*). Further studies are needed to determine which diabetic mouse model is the most appropriate model of melioidosis in humans with diabetes and to evaluate potential candidate vaccines in that model.

### Nonhuman Primate Model

It is currently unclear which NHP model is the most suitable for melioidosis vaccine evaluation. Only the marmoset has been evaluated by using a *B. pseudomallei* subcutaneous inoculation route (*23*). On the basis of data obtained in melioidosis inhalation models, the marmoset appears to be the most susceptible NHP; the rhesus macaque (*Macaca mulatta*) is of intermediate susceptibility (*26*), and the cynomolgus macaque (*M. fascicularis*) is the most resistant to *B. pseudomallei* inoculation (unpub. data). The African green monkey (*Chlorocebus aethiops*) is reported to be comparable to the rhesus macaque in terms of susceptibility to *B. pseudomallei* when inoculated by using the inhalation route (*27*). Further studies are needed to determine which NHP model is the most representative of human melioidosis and will be most useful for evaluation of candidate vaccines.

### Immunity among Persons with Diabetes

Immunity and response to vaccines in humans with diabetes requires further study. In the cases of influenza and hepatitis B vaccines, persons who have diabetes have comparable responses to those who do not, and persons with diabetes acquire protection against these viruses (*28*–*31*). Effectiveness of pneumococcal vaccines is also observed in persons with diabetes, particularly in preventing pneumococcal bacteremia (*32*,*33*). However, the immune deficit leading to susceptibility to melioidosis in persons with diabetes has not been clearly established. The mechanisms of immune protection against melioidosis are complex and involve both innate and adaptive immunity (*3*). An understanding of immunologic responses among persons with diabetes against melioidosis and correlates of protection would inform the development of a melioidosis vaccine that protects against natural infection.

## Funding for Head-to-Head Comparison and Future Objectives of SGMVD

The SGMVD will seek funding from multiple sources for the head-to-head comparison of candidate vaccines at an independent institute. The SGMVD aims to communicate by using email, meet yearly to steer the process of vaccine development, continue to identify knowledge gaps on vaccine development, and ensure that the head-to-head comparison is implemented and conducted within a timely fashion.

### Potential of Phase I, II and III Clinical Studies

Using the stage-gating approach, the SGMVD will develop robust criteria for advancing vaccine candidates from the NHP model stage to the phase I, II, and III clinical studies. SGMVD will also consider vaccine candidates developed for biodefense purposes if any are found to be effective by other groups, including Defense Threat Reduction Agency, National Institute of Allergy and Infectious Disease, and BARDA. On the basis of preliminary discussions, candidate vaccines that pass the first head-to-head comparison in 2 different mouse models and a further diabetic mouse model should go forward to be evaluated in a NHP model. The SGMVD does not consider that a diabetic NHP model is required. The first phase I and phase II human clinical studies should initially be conducted in healthy volunteers to determine safety and immunogenicity of vaccines, and promising candidates should be examined in phase I and phase II clinical studies that include persons with diabetes.

## Conclusions

The SGMVD has recognized the need for the head-to-head comparison of candidate vaccines in standardized mouse models using a defined set of criteria and an independent institute to evaluate melioidosis candidate vaccines for advancement to the NHP model stage. None of the animal models will be a perfect reflection of human infection; the main purpose of using mouse and NHP models is to carefully select the optimal candidate vaccines for future clinical studies by using a gating process approach.

The ultimate aim of the SGMVD is to facilitate the production of a vaccine to protect against naturally acquired infection, with potential to be used in high-risk groups such as persons with diabetes in areas to which melioidosis is endemic, such as northeast Thailand. The SGMVD has identified knowledge gaps in the process of advancing melioidosis vaccines to the point of early phase clinical studies. The SGMVD will also continue to seek advice from experts in related fields over time, including those with expertise in vaccine development and testing, animal models (*20*,*21*), and bacterial genomics. This committee aims to hasten the processes required to obtain a cost-effective, safe, and licensed vaccine against melioidosis.

**Technical Appendix.** Questionnaire for members of the Steering Group on Melioidosis Vaccine Development in preparation for a meeting of international candidate vaccine developers, researchers, and government health officials that was held in March 2014 to reach consensus of opinions and recommendations on the development of vaccines against naturally acquired melioidosis.
