# Supplementary material for: Consensus on the Development of Vaccines against Naturally Acquired Melioidosis
Source: Emerg Infect Dis. 2015 Jun;21(6):e141480. doi: 10.3201/eid2106.141480 (PMC4451926; doi:10.3201/eid2106.141480)
Supplement: Supplementary file 1 — Technical Appendix. Questionnaire for members of the Steering Group on Melioidosis Vaccine Development in preparation for a meeting of international candidate vaccine developers, researchers, and government health officials that was held in March 2014 to reach consensus of opinions and recommendations on the development of vaccines against naturally acquired melioidosis. [file 14-1480-Techapp-s1.pdf]

Article DOI: http://dx.doi.org/10.3201/eid2106.141480

# Consensus on the Development of Vaccines against Naturally Acquired Melioidosis

## Technical Appendix

## Questionnaire on Melioidosis Vaccine Development

**1. Key points**

1.1 There are three possible routes of *B. pseudomallei* infection (inhalation, inoculation and ingestion), and a melioidosis vaccine will need to provide protection against multiple routes of infection.

1.2 Protective and therapeutic vaccines are both needed, and candidates should be evaluated for both indications.

1.3 Although subunit vaccines would be predicted to have the highest safety profile, optimal candidates may not be developed and ready for testing in Phase I studies for another 5 to 10 years. In the meantime, the best available vaccine candidates (either live, whole cell killed or subunit) should be moved forward to non-human primate models and Phase I trials.

1.4 Heat killed *B. pseudomallei* vaccine with CLDC adjuvant shows the highest efficacy in mice models following inhalation challenge thus far (*1*). Does the optimal adjuvant require more investigation prior to testing in non-human primate models?

1.5 Live attenuated *B. pseudomallei* may not be an option because the organism has a very well documented ability to become latent in the human host for many years. This means that safety trials would need extensive follow up before concluding that a live attenuated vaccine was safe. This may rule out even a highly attenuated strain (please comment if you agree).

1.6 Live *B. thailandensis* would be predicted to have a safer safety profile, and the study using *B. thailandensis* variant which presents *B. pseudomallei*-like CPS (strain E555) has been evaluated as a vaccine candidate in mice with promising preliminary results (*2*). In view of this, further animal experimental work in mice is warranted to determine protection from live *B. thailandensis* in comparison with heat-killed *B. pseudomallei* plus the optimal adjuvant.

**Comments from Melioidosis Vaccine Working Committee**
**Name:**

**Comments for point 1, including 1.5:**

**2. Available vaccine candidates**

2.1 The best vaccine candidate for inhalational challenge based on literature evidence is heat killed *B. pseudomallei* with CLDC adjuvant evaluated by Angela Henderson (1st author), Steven Dow (last author), *et al* in BALB/c mice immunized via mucosal route and challenged with inhalation of *B. pseudomallei* 1026b strain (7500 cfu or about 40 MLD [Median Lethal Dose]) at Colorado State University, US (*1*). All mice survived up to day 40, and half survived up to day 60.

The heat-killed *B. pseudomallei* without adjuvant evaluated by Sarkar-Tyson (1st author), Richard Titball (last author), *et al* in BALB/c mice immunized via intraperitoneal route and challenged with inhalation of *B. pseudomallei* K96243 strain (92 cfu or about 18 MLD) (*3*). Median time to death ranged from 8 to 9.75 days, compared to 5.86 days in control group.

2.2 The best vaccine candidate for inoculation challenge based on literature evidence is E555 Live *B. thailandensis*, which has been evaluated by Andrew Scott (1st author), Tim Atkins (last author), *et al* in the BALB/c mice model challenged with intraperitoneal inoculation of *B. pseudomallei* K96243 strain ($10^6$ cfu or about 6,000 MLD) at DSTL, UK (manuscript in preparation). All mice survived up to day 42.

2.3 No candidates have been tested in an ingestion challenge model.

| **Comments for point 2:** |
|---|
| |

**3. Non-human primate (NHP) models**

3.1 There are currently three NHP models for inhalational challenge

3.1.1) USAMRIID, Rhesus Macaques and African Green Monkey (*4*)

3.1.2) DSTL, Marmoset (*5*)

3.1.3) PHE, Rhesus (unpublished)

3.1.4) Tulane Primate Center, Macaques (unpublished)

3.2 There are no NHP models for inoculation challenge

3.3 There are no NHP models for ingestion challenge

**Comments for point 3:**

**4. Prioritisation of the needs**

4.1 The strongest need is for a vaccine that can prevent infection via the inoculation route in melioidosis endemic areas. This represents the first aim of the proposal.

4.2 There is a strong need for a vaccine that can be used after inhalation or suspected inhalation of *B. pseudomallei* that results from a deliberate release in melioidosis non-endemic areas. This represents the second aim of the proposal.

**Comments for point 4:**

**5. Objectives**

**5.1 Select the best candidates for NPH by comparing multiple candidates (heat-killed *B. ps* with live E555, and with subunit vaccine (if readily available to be evaluated)) in an inoculation mice model**

Model: BALB/c mice
Immunization route: intradermal inoculation
Study arms: (1) heat killed K96243 *B. ps* with CLDC adjuvant
(2) heat killed K96243 *B. ps* with alternative adjuvant?
(3) heat killed K96243
(4) CLDC
(5) E555
(6) PBS as negative control
(7) other candidates
Time of immunization: (1) prior to challenge
(2) after challenge for 2 days (48-hours)
Strain challenge: K96243
Route of challenge: intradermal inoculation
Dose of challenge: 40 MLD
Duration of observation: 30 days
Outcome measurement: 1) Mortality rate
2) Hazard ration at least 0.5
3) Survive the 1st week after onset of symptoms

**Comments for point 5.1:**

**5.2 Best vaccine candidates (can be up to 3 candidates) in murine inoculation model in 5.1 should be evaluated in NHP inoculation model**

Model: NHP
Immunization route: intradermal inoculation
Study arms: best 3 candidates from 5.1
Time of immunization: (1) prior to challenge
(2) after challenge for 2 days (48-hours)
Strain challenge: K96243
Route of challenge: intradermal / subcutaneous inoculation
Dose of challenge: 40 MLD
Outcome measurement: 1) Mortality rate
2) Hazard ration at least 0.5
3) Survive the 1st week after onset of symptoms

**Comments for point 5.2:**

**5.3 Best vaccine candidate(s) in NHP inoculation model should be evaluated for cross protection to inhalation model.**

**Comments for point 5.3:**

**The following questions are to try to set up our F2F meeting by the end of March 2014 if possible.**
**(We don't have a source of funding yet, but would like to ask and see the total and we will search from multiple sources if possible)**

**Are you going to attend ASM biodefense between 27-29 January 2014 in Washington DC ?**
Yes / No / Maybe

**Are you able to come to UK for a meeting at PHE, UK on any day between 17 to 28 March 2014 ?**
Yes, I am available on 17, 18, 19, 20, 21, 24, 25, 26, 27, 28 / No, I am not available on those days

**Do you need a funding for transportation / hotels if it's set up at PHE, UK ?**
Yes, I need a funding of about x,xxx pounds / No

**Do you have any suggestion for source of funding ?**
Yes / No

**Other comments:**

## References

1. Henderson A, Propst K, Kedl R, Dow S. Mucosal immunization with liposome-nucleic acid adjuvants generates effective humoral and cellular immunity. Vaccine. 2011; 29:5304–12. http://dx.doi.org/10.1016/j.vaccine.2011.05.009

2. Scott AE, Laws TR, D'Elia RV, et al. Protection against experimental melioidosis following immunization with live *Burkholderia thailandensis* expressing a manno-heptose capsule. Clinical and vaccine immunology. 2013; 20:1041–7. http://dx.doi.org/10.1128/CVI.00113-13

3. Sarkar-Tyson M, Smither SJ, Harding SV, Atkins TP, Titball RW. Protective efficacy of heat-inactivated *B. thailandensis, B. mallei* or *B. pseudomallei* against experimental melioidosis and glanders. Vaccine. 2009; 27:4447–51. PubMed http://dx.doi.org/10.1016/j.vaccine.2009.05.040

4. Yeager JJ, Facemire P, Dabisch PA, Robinson CG, Nyakiti D, Beck K, et al. Natural history of inhalation melioidosis in rhesus macaques (*Macaca mulatta*) and African green monkeys (*Chlorocebus aethiops*). Infect Immun. 2012;80:3332–40. PubMed http://dx.doi.org/10.1128/IAI.00675-12.

5. Nelson M, Dean RE, Salguero FJ, et al. Development of an acute model of inhalational melioidosis in the common marmoset (*Callithrix jacchus*). International journal of experimental pathology. 2011; 92(6):428–35.
